# A Broader Perspective on the Prenatal Diagnosis of Cornelia de Lange Syndrome: Review of the Literature and Case Presentation

**DOI:** 10.3390/diagnostics11010142

**Published:** 2021-01-19

**Authors:** Anca Maria Panaitescu, Simona Duta, Nicolae Gica, Radu Botezatu, Florina Nedelea, Gheorghe Peltecu, Alina Veduta

**Affiliations:** 1 Department of Obstetrics and Gynecology, Carol Davila University of Medicine and Pharmacy, 020021 Bucharest, Romania; gica.nicolae@umfcd.ro (N.G.); radu.botezatu@umfcd.ro (R.B.); ina.nedelea@gmail.com (F.N.); gheorghe.peltecu@umfcd.ro (G.P.); 2 Filantropia Clinical Hospital, 011171 Bucharest, Romania; simona_duta@yahoo.com (S.D.); alina.veduta@gmail.com (A.V.); 3 Department of Genetics, Carol Davila University of Medicine and Pharmacy, 020021 Bucharest, Romania

**Keywords:** Cornelia de Lange, genetic syndrome, prenatal diagnosis, ultrasound

## Abstract

Cornelia de Lange syndrome (CDLS) is caused by pathogenic variants in genes which are structural or regulatory components of the cohesin complex. The classical Cornelia de Lange (CDLS) phenotype is characterized by distinctive facial features, growth retardation, upper limb reduction defects, hirsutism, and developmental delay. Non-classical phenotypes make this condition heterogeneous. Although CDLS is a heterogeneous clinical and genetic condition, clear diagnostic criteria have been described by specialist consensus. Many of these criteria refer to features that can be seen on prenatal ultrasound. The aim of this paper is twofold: to present the ultrasound findings in fetuses affected by CDLS syndrome; to discuss the recent advances and the limitations in the ultrasound and genetic prenatal diagnosis of CDLS. Our review aims to offer, apart from the data needed to understand the genetics and the prenatal presentation of the disease, a joint perspective of the two specialists involved in the prenatal management of this pathology: the fetal medicine specialist and the geneticist. To better illustrate the data presented, we also include a representative clinical case.

## 1. Introduction

Corelia de Lange syndrome (CDLS) which, in typical cases, is easily recognizable is a genetic disease (OMIM entries 122470, 300040, 300269, 300590, 300882, 606062, 606462, 608667, 610759, and 614701) with heterogeneous clinical presentation and many possible causal genes [1,2]. The proteins encoded by genes involved in CDLS are all structural or regulatory components of the cohesin complex [1,3]. Overall, the CDLS phenotype can be characterized as a spectrum to which the classic CDLS phenotype belongs as well as similar but non-classic phenotypes caused by pathogenic variants in genes involved in cohesin functioning [1,2,3,4,5,6,7].

The typical Cornelia de Lange (CDLS) phenotype is characterized by distinctive craniofacial appearance, growth retardation, reductional limb defects (mostly of the upper limbs), hirsutism, and neurocognitive delay [1,2,3,4,5,6,7]. The typical facial features in CDLS consist of synophrys, long eyelashes, depressed nasal bridge, short nose with anteverted nares, long philtrum, thin lips, downward turning corners of the mouth, and micrognathia. Severe gastroesophageal reflux (gastroesophageal reflux disease, GERD) can be a significant problem in individuals with CDLS. Congenital diaphragmatic hernia as well as palatal, cardiac and genitourinary malformations are frequent but not typical findings in CDLS [1,2,3,4,5,6,7].

The prevalence of CDLS is estimated to be between 1 in 10,000 and 1 in 30,000 live births. The prevalence is probably underestimated, as mild cases are likely to not be reported [1,7,8].

As CDLS is a rare disease, the pediatric literature mainly consists of small to medium-size case series [5,9,10,11,12,13,14,15,16,17]. Reviews of the knowledge available to the date were published in 2015 and 2020 [1,6,7].

In 2018, pediatricians published a consensus on CDLS diagnosis [2]. This document acknowledges the heterogeneity that exists in diagnostic approaches and care practices for CDLS patients and the need to develop better scores for the disease severity, preferably stratified by genetic cause [2]. The 2018 consensus classifies the clinical diagnostic criteria for CDLS into cardinal features and suggestive features. According to the consensus, the cardinal features are synophrys and/or thick eyebrows, short nose, concave nasal ridge and/or upturned nasal tip, long and/or smooth philtrum, thin upper lip vermilion and/or downturned corners of the mouth, hand oligodactyly and/or adactyly, and congenital diaphragmatic hernia. Suggestive features for CDLS include global developmental delay and/or intellectual disability, prenatal growth retardation, postnatal growth retardation, microcephaly (prenatally and/or postnatally), small hands and/or feet, short fifth finger, and hirsutism (Table 1). A score of two points is assigned to each cardinal feature, if present and a score of one point is assigned to each suggestive feature. A score of ≥11 points indicates classic CDLS, if at least three cardinal features are present; a score of 9–10 points indicates non-classic CDLS, if at least two cardinal features are present; a score of ≥4 points is sufficient to warrant molecular testing for CDLS, if at least one cardinal feature is present; a score below four points is insufficient to indicate testing for CDLS [2]. The consensus states that a score of ≥11 points confirms the diagnosis of CDLS regardless of whether a pathogenic variant in one of the known genes can be found; it means that the diagnosis of CDLS remains clinical.

While the literature on the postnatal diagnosis of CDLS is nowadays quite ample, well analyzed, and sufficient to guide clinical practice, literature on the prenatal diagnosis of CDLS is not extensive. This is actually not surprising as CDLS is a rare genetic syndrome and rare genetic syndromes are generally considered not amenable to systematic prenatal diagnosis. Despite the fact that prenatal diagnosis does not specifically target rare genetic syndromes, a small number of reports of cases and series of parentally detected CDLS cases have been sporadically published in the last decades [18,19,20,21,23,24,25,26,27,28,29,30,31,32,33,34,35,36,37,38].

A summary of the data available on the prenatal diagnosis of CDLS might be useful to the fetal medicine practitioner. Our aim is twofold: to present the ultrasound findings in fetuses affected by CDLS syndrome; to discuss the recent advances and the limitations in the ultrasound and genetic prenatal diagnosis of CDLS. The review aims to offer, apart from the data needed to understand the genetics and the prenatal presentation of the disease, a broader perspective of the two specialists involved in the prenatal management of this pathology: the fetal medicine specialist and the geneticist. To better illustrate the data presented, we also include a representative clinical case.

While postnatal diagnosis of CDLS is mainly clinical, the examination of the fetal phenotype by ultrasound can only raise suspicion of a genetic syndrome, prenatally. Therefore, the role of the genetic diagnosis is actually different, prenatally, and postnatally. While postnatal genetic analysis only confirms the clinical diagnosis and helps the differential diagnosis in non-classical cases, the main role of prenatal genetic testing would be to prove the diagnosis in cases where fetal ultrasound shows a classical CDLS phenotype. Genetic confirmation of a fetal condition is substantially informative and enlarges the range of options available to families. On the other hand, molecular genetic testing has serious practical and even ethical limitations, in the prenatal setting, in cases caused by de novo mutations. Variants of unknown significance (VOUS) are an issue with molecular genetic testing in general, and a particularly difficult challenge, prenatally [39,40]. A geneticist’s opinion on the genetic prenatal diagnosis of CDLS is included in the text of the article.

Ultimately, prenatal diagnosis could improve the prognosis of individuals affected by CDLS, by optimizing perinatal care. The severe feeding problems of newborns with CDLS should be addressed by experienced neonatologists, right after birth. Many of the health problems of CDLS babies can become neonatal emergencies, including surgical emergencies [1,2].

## 2. Genetics of CDLS and Prenatal Genetic Testing for CDLS

Even in the context of next generation sequencing techniques (NGS), molecular prenatal testing in cases caused by de novo mutations have been considered rarely effective, until recently. This situation is rapidly changing nowadays. In the particular circumstances of CDLS, as the fetal appearance in classical CDLS is highly suggestive, ultrasound could be able to select cases for genetic testing if the sonographer is theoretically aware of the disease. On the other hand, the molecular diagnosis of CDLS is complicated by the presence of somatic mosaicism and by the overlap with other diseases [2,41,42]. A geneticist’s opinion on practical issues of the genetic prenatal diagnosis of CDLS is included in the text of the article.

Seven genes associated with CDLS have been identified: nipped-B like protein (*NIPBL*), structural maintenance of chromosomes 1A (*SMC1A*) and 3 (*SMC3*), double-strand break repair protein rad21 homolog (*RAD21*), bromodomain-containing protein 4 (*BRD4*), histone deacetylase 8 (*HDAC8*), and ankyrin repeat domain-containing protein 11 (*ANKRD11*) [1,2,6,7,42,43,44,45]. Most individuals with CDLS have either a heterozygous pathogenic variant in *NIPBL*, *RAD21*, or *SMC3* or a hemizygous pathogenic variant in *HDAC8* or *SMC1A*. The most prevalent mutations are in the *NIPBL* gene, found in 60–70% of affected individuals. Most of the affected individuals have a de novo pathogenic variant. Less than 1% of individuals with *NIPBL*-related CDLS have an affected parent. When the parents are clinically unaffected, the risk to the siblings of a proband with *NIPBL*-related CDLS is estimated to be 1.5% because of the possibility of germline mosaicism [1,2,42,43,44,45].

All proteins encoded by genes known to be associated with CDLS belong to the cohesin complex [2,3,46,47,48]. The cohesin complex, composed of four core subunits, SMC1A, SMC3, RAD21 and STAG, is evolutionarily conserved and has an important role in chromatid cohesion, gene expression regulation and DNA repair [1,2,3,47,48]. Additional factors, such as NIPBL and HDAC8, regulate the activity of cohesin during the cell cycle. Cohesin loading onto chromatin is mediated by NIPBL, together with the partner molecule MAU2. It occurs in G1 stage in yeast or at the end of telophase in mammalian cells [1,2,3,47,48]. Cohesin was first identified for its role in establishing sister chromatid cohesion, which is important for proper chromosome segregation, but cohesin is also a key regulator of gene expression. Cohesin, together with its loader NIPBL and with the sequence-specific DNA binding protein CTCF (CCCTC-binding factor insulator protein), cooperate to organize the genome into structural topologically associated domains (TADs), chromatin loops, and contact domains. It therefore brings together distant enhancers with promoter sequences to orchestrate gene expression [3,46,47,48].

Cells from CDLS patients do not show sister chromatid cohesion defects suggesting that the pathogenesis of CDLS is not directly linked to the disruption of sister chromatid cohesion but to inability of the complex to regulate gene expression. CDLS cells show genome instability, likely as a result of reduced DNA repair capability [3,46,47,48].

Mutations in *NIPBL* (nipped-B-like protein, 5p13.2) are associated with the classical CDLS phenotype, in an apparently dose-dependent manner [3,48,49,50].

Mutations in *RAD21* (double-strand-break repair protein rad21 homolog, 8q24.11) have been rarely observed in association with a non-classic CDLS phenotype featuring structural anomalies consistent with CLDS but only minor to mild developmental delay [51,52,53].

Mutations in *SMC3* (structural maintenance of chromosomes 3, 10q25.2) are associated with atypical CDLS forms featuring some degree of cognitive delay but only mild facial dysmorphism and no absence or reduction of limbs or digits [54,55].

Mutations in *BRD4* (bromodomain-containing protein 4) are considered too rare to enable conclusions on the most common phenotype. The pathogenic mechanism might involve sequestration of NIPBL by the abnormal gene product [56].

Mutations in *ANKRD11* (ankyrin repeat domain-containing protein 11) are associated with a non-classic/overlapping CDLS phenotype [57].

Mutations in *HDAC8* (histone deacetylase 8, Xq13.1, can be inactivated) generate a remarkably variable phenotype, with some individuals fulfilling the criteria for classic CDLS [58,59,60].

Mutations in *SMC1A* (structural maintenance of chromosomes 1A, Xp11.22, not inactivated) are the second most common mutations known to be involved in CDLS. They are associated with a non-classic phenotype with predominant mental retardation, which can vary from mild to severe [54,61,62,63].

Recently, mutations in other genes (*EP300*, *TAF6*) have been shown to cause CDLS–overlapping phenotypes [64,65].

## 3. Ultrasound Prenatal Diagnosis of CDLS

Postnatal diagnosis of CDLS is clinical, based on the observed features of the phenotype. A completely similar approach is obviously not possible prenatally, but high-resolution ultrasound can depict fetal anatomy in detail. Awareness of a rare disease, on the other hand, cannot be expected from the average sonographer.

Based on our own experience and on a review of the literature, we aim to systematically present the features that can be identifiable by ultrasound and could lead to the prenatal suspicion/diagnosis of CDLS. A similar work was undertaken by Pajkrt et al. in 2010 [31] and by Avagliano et al. in 2017 [22] but the pediatric consensus that describes and classifies the clinical diagnostic criteria for CDLS was not available then. We analyze the possibility of prenatal ultrasound detection of the cardinal and suggestive features of CDLS, which were described by the 2018 consensus on CDLS (Table 1). This information can be used for the prenatal diagnosis of classical CDLS and for a systematic diagnostic approach in cases of growth restricted fetuses with structural anomalies, in general.

Cardinal and suggestive features that make prenatal diagnosis possible include thick eyebrows which meet at midline (synophrys); short nose with depressed nasal ridge and anteverted nares; long and smooth philtrum; thin upper lip and downturned corners of mouth; hand oligodactyly or adactyly; congenital diaphragmatic hernia; fetal growth restriction; microcephaly; and short fifth finger [2,22,31].

The distinctive facial features of CDLS (synophrys; short nose with depressed nasal ridge and anteverted nares; long and smooth philtrum; and thin upper lip and downturned corners of mouth) can be seen on fetal ultrasound [18,19,20,21,23,24,25,26,27,28,29,30,31,32], especially if volumetric rendering is used [19,20]. Even in the absence of 3D reconstruction, detailed ultrasound examination of the fetal face in a midsagittal view can demonstrate the abnormal appearance of the nose and philtrum. The first reports describing CDLS facial features of fetuses date from the 1990s, when the resolution of ultrasound machines was less high and volume technology was not available [18,23,24]. Synophrys might be detected by 2D ultrasound but is better seen on 3D ultrasound [18,19,20,21].

Severe limb defects should be always diagnosed on prenatal ultrasound. Mild and even subtle ones too are detectable by detailed ultrasound examination. Upper limb defects in CDLS can be unilateral or bilateral, if bilateral they are usually asymmetrical, and they range from clinodactyly to complete absence of the limb. Oligodactyly and ectrodactyly are frequent in CDLS. Limb reduction defects can guide the diagnosis toward CDLS, in cases of fetal growth restriction [20,21,22,30,31,32].

Congenital diaphragmatic hernia is a major structural malformation which can be diagnosed prenatally in most cases [28]. The 2018 pediatric consensus classifies it as a cardinal feature of CDLS [2]. In fact, congenital diaphragmatic hernia can be seen in many fetal genetic syndromes; the differential diagnosis in cases of growth restricted fetuses with diaphragmatic hernia is large [32].

Growth restriction (including microcephaly) can be diagnosed by prenatal ultrasound. The failure to thrive seen in individuals affected by CDLS is partially due to their severe feeding difficulties, but the onset of growth restriction is prenatal, in CDLS [18,23,24,33]. Fetal growth restriction was documented in virtually all cases where CDLS was diagnosed in utero [18,19,20,21,23,24,25,26,27,28,29,30,31,32,33,34,35,36,37,38]. The growth restriction typically worsens with time in fetuses with CDLS, but placental and fetal Doppler flows remain normal throughout pregnancy [18,22,23,24,33].

A short fifth finger can be seen on prenatal ultrasound. For a long while, clinodactyly was looked for on fetal ultrasound because it was considered a sign for trisomy 21 (Down’s syndrome), but the specificity and the sensitivity of the finding are low. It is likely that the short fifth finger in CLDS is caused by an anomaly of the metacarpal bones and not by absence of the middle phalanx as in trisomy 21 [21,22].

Other ultrasound findings, besides the diagnostic criteria listed by the 2018 pediatric consensus, are reported in fetuses with CDLS.

There are reports of increased nuchal translucency in the first trimester, in fetuses with CDLS [27].

Long eyelashes can provide a clue to the diagnosis in fetuses displaying other CDLS features [36].

The profile of the fetuses affected by CDLS is abnormal not only because of the features listed as clinical diagnosis criteria but also because of micrognathia [21,22,32]. Micrognathia is a structural malformation usually amenable to prenatal diagnosis. Facial clefts complicate the postnatal evolution in CDLS but are not specific [2,32].

Cardiac malformations are often seen in fetuses with CDLS [2,32], although one relatively recent paper stated otherwise [22]. Ultrasound is useful to diagnose fetal heart anomalies, but the finding of a cardiac malformation is not specific and the differential diagnosis in cases of growth restricted fetuses with heart defects is large [31,32]. Prenatal diagnosis of heart defects is important in terms of fetal prognosis and perinatal management.

In the end of the discussion on the ultrasound prenatal diagnosis of CDLS, we should state the role of volumetric ultrasound in detecting the distinctive phenotype of classical CDLS.

## 4. Prenatal Presentation of a Classical CDLS Case

**Figure 1:**
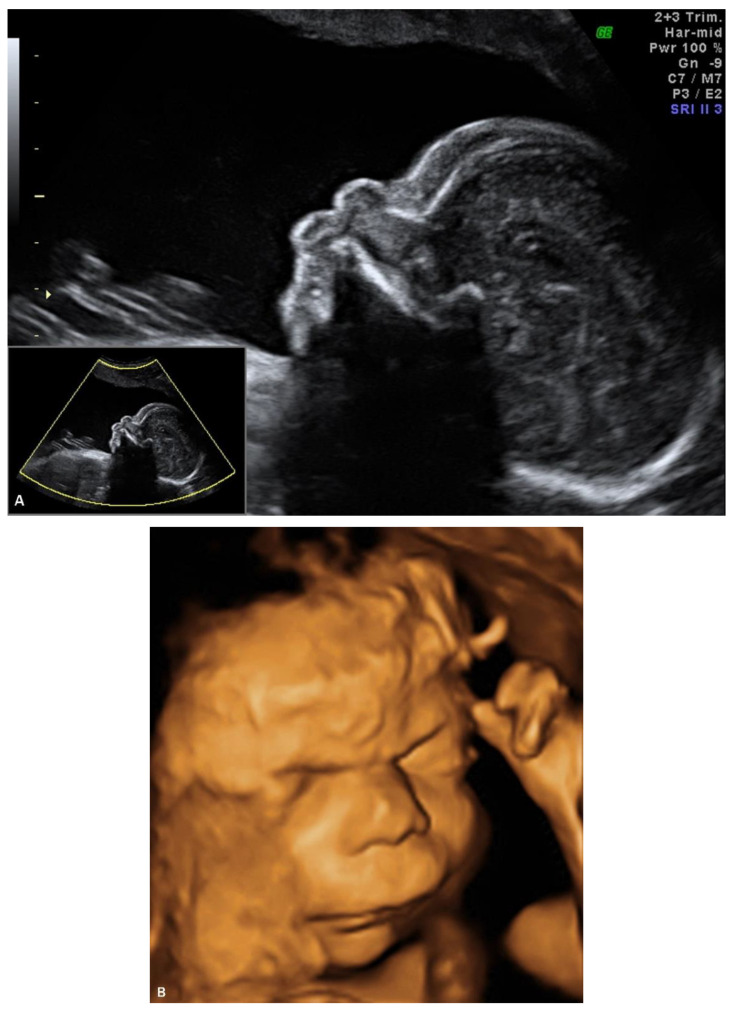

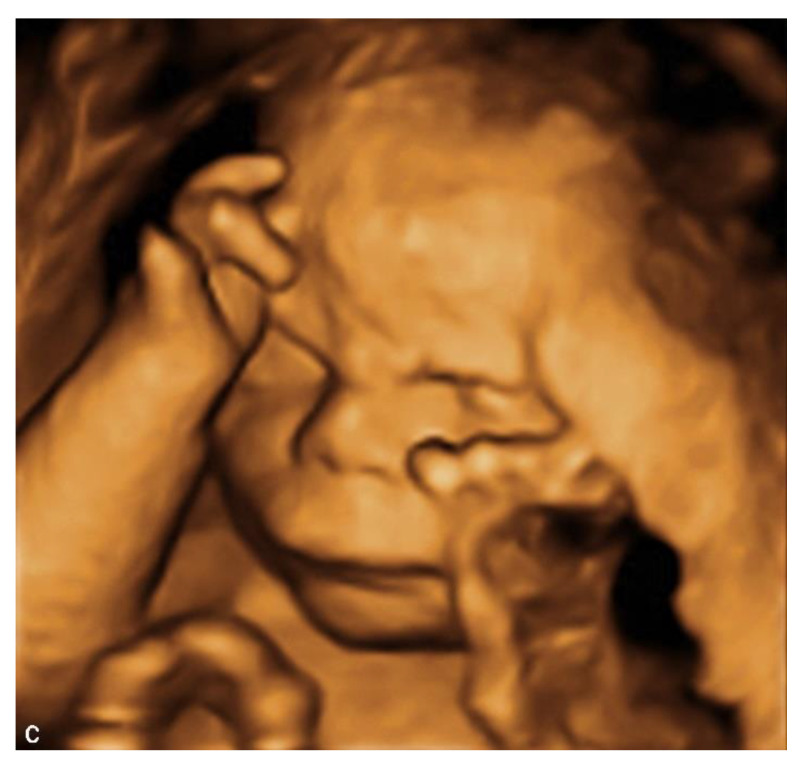
Ultrasound examination of the fetal face in a CDLS case with classical phenotype: (**A**) 2D image of the fetal profile at 29 w of gestation, showing mild micrognathia/retrognathia; abnormal philtrum; depressed nasal bridge; (**B**) Volume reconstruction of the fetal face at 29 w of gestation; (**C**) Volume reconstruction of the fetal face at 34 w of gestation.

We report a case of prenatal diagnosis of CDLS at 29 weeks’ gestation in a 30-year-old woman who had previously delivered a normal baby. The patient was referred to our fetal medicine unit because of fetal growth restriction, diagnosed at 24 weeks’ gestation. As no Doppler flow anomalies were detected, a genetic anomaly (lethal trisomy) rather than placental insufficiency was suspected by the referring doctors. Ultrasound examination in our unit demonstrated symmetrical fetal growth restriction with normal Doppler studies. The amniotic fluid was increased with a deepest vertical pool of 9.2 cm. Examination of the fetal face showed abnormal profile, synophrys, depressed nasal bridge, long downturned upper lip, and micrognathia (Figure 1A,B). Cardiac examination demonstrated abnormal flow through the atrio-ventricular, aortic, and pulmonary valves. The third finger of the right fetal hand was missing, and the fingers of the left hand were overlapping.

The suspected diagnosis was Cornelia de Lange syndrome, CDLS. We discussed the findings with the patient, and we presented the parents with the choice of a late amniocentesis. We explained that lethal conditions such as trisomy 18 or 13 can be ruled out only by direct genetic testing. We described the genetic tests that can be performed for CDLS and we discussed the limitations of such tests, given the circumstances. A geneticist was involved in the counselling. The parents refused any prenatal genetic testing, as they were committed to the pregnancy.

The initial ultrasound findings persisted in the third trimester (Figure 1C). As pregnancy progressed the polyhidramnios worsened but did not become tense and did not require amniodrainage. Fetal skin edema was seen in late pregnancy.

A male infant weighing 2030 g was delivered by cesarean section at 38 weeks of gestation. An experienced neonatologist was present at birth. CDLS was confirmed by clinical examination demonstrating synophrys, long eyelashes, short upturned nose, thin downturned lips, long philtrum, excessive body hair, mild aortic valve stenosis and moderate aortic arch hypoplasia, missing fingers, and severe gastroesophageal reflux. A multidisciplinary team, including a geneticist, was involved in the care of the baby. Genetic testing was offered after birth, but it was declined by the parents of the baby.

**Figure 2:**
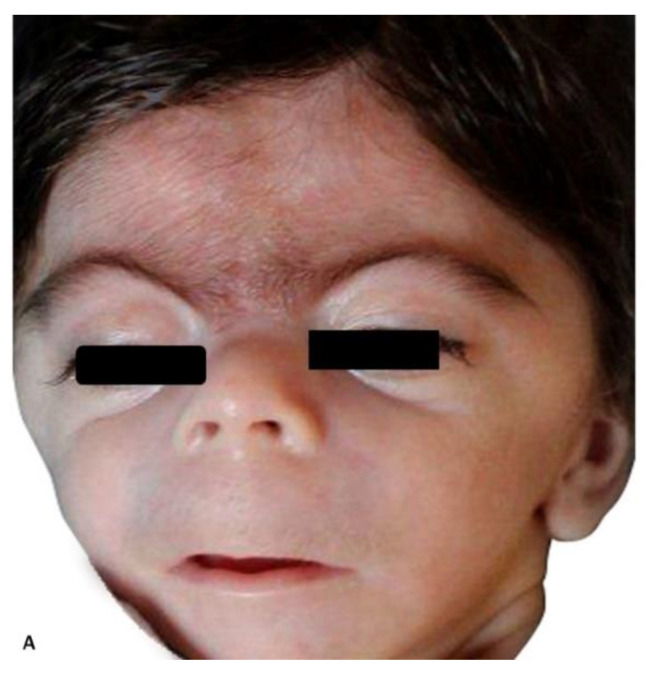

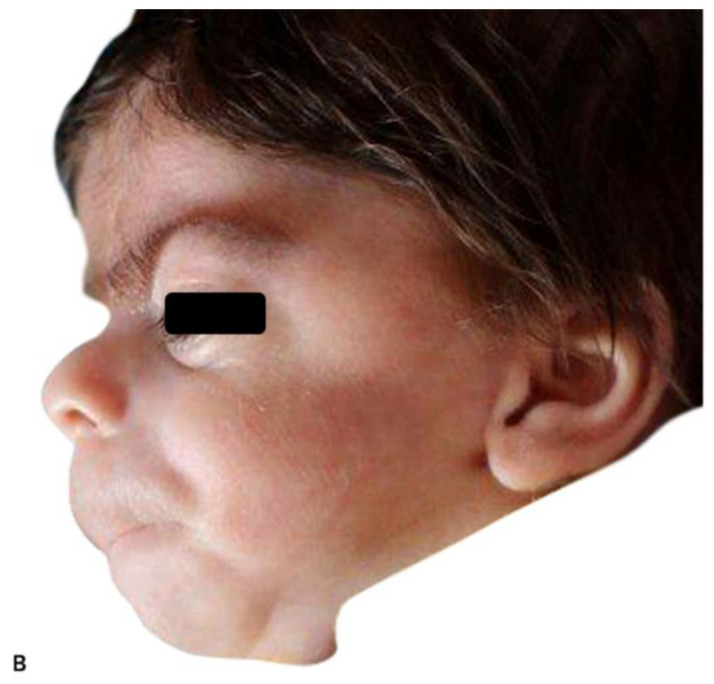
Postnatal images of the patient depicted in Figure 1: (**A**) frontal view of the face; (**B**) profile view of the face.

The postnatal pictures of the infant show striking resemblance to the prenatal volumetric reconstruction of the face (Figure 2A,B). Volumetric rendering of the fetal face was an important component of the diagnostic approach, in this case.

The case presentation emphasizes the role of 3D ultrasound in the prenatal detection of CDLS.

## 5. Discussion

### 5.1. The Geneticist’s Point of View

It is appropriate to consider prenatal genetic testing in high risk pregnancies, defined after ultrasound identification of fetal structural anomalies. Specialized multidisciplinary pretest counselling, by a geneticist and a feto-maternal specialist, is as important as testing itself. This is a particularly important issue in Cornelia de Lange, due to known limitations in genetic testing for CDLS, including somatic mosaicism that cannot be always detected. Not all CDLS cases have a genetic cause that can be identified in the present. Therefore, parents should be aware that a “negative” genetic result is not always reassuring and cannot exclude the possibility of genetic etiology.

Depending on the gestational age and associated features, different prenatal testing approaches could be considered. Thus, in the first trimester, for the fetuses with increased NT (>P95), chorionic villus biopsy with microarray analysis could be the first option, because of the lack of specificity of this ultrasound finding and the relatively high frequency of aneuploidies, in those situations. If the result is normal, and on subsequent ultrasound monitoring other fetal structural anomalies become obvious, further testing with exome analysis (Whole Exome Sequencing, WES) based on NGS technologies is preferable. A maybe better solution is simultaneous analysis microarray and exome testing, ideally trio exome (fetus plus parents) in order to optimize the time until final diagnosis. Nevertheless, even in the case of genome-wide approach, good ultrasound documentation of the CDLS suspicion is advisable, in order to increase the possibility to identify the genetic cause.

In the second trimester, in cases with high suspicion of CDLS, based on “facial recognition” and associated malformations, a multigene panel for CDLS could be an option. If the differential diagnosis is broad, genome wide approach (WES) with simultaneous CNV analysis could be advisable.

In cases with classical phenotype, after genetic counselling that includes a discussion on ethical issues in cases of “negative results”, multigene panel testing of *NIPBL*, *SMC1A*, *SMC3*, *HDAC8*, and *RAD21* genes would be the more cost effective and less time-consuming method. If this test has a negative result, deletion and duplication testing of *NIPBL* gene can be performed using multiplex ligation-dependent probe amplification (MLPA) or chromosome microarray.

In our opinion, because of the difficulty of a specific prenatal diagnosis, based only on ultrasound features, it is advisable to add the CDLS genes *NIPBL*, *SMC1A*, *SMC3*, *HDAC8*, and *RAD21* in broad multigene panels for fetuses with growth restriction and any structural anomalies.

### 5.2. The Fetal Medicine Specialist’s Point of View

Our opinion is that classical CDLS can be detected prenatally if the disease is sought out and volumetric ultrasound rendering is used. The resolution of fetal ultrasound machines is nowadays high, and the fetal appearance is very suggestive for the diagnosis, in classical CDLS. Instead, awareness of a rare disease is difficult to be achieved and cannot be expected from the average sonographer. The solution could be a well-defined system of referring cases of fetuses with growth restriction and any structural malformations to centers where fetal medicine specialists and geneticists involved in prenatal diagnosis are available. In some instances, features of CDLS could even be seen as early as the first trimester and therefore prompt referral could lead to earlier diagnosis [66].

## Figures and Tables

**Table 1:** Prenatal ultrasound diagnosis of cardinal and suggestive features of Cornelia de Lange Syndrome.

Prenatal Ultrasound Diagnosis	
Cardinal Features *	
Synophrys and/or thick eyebrows	Possible, especially with volumetric ultrasound [18,19,20,21,22]
Short nose, concave nasal ridge and/or upturned nasal tip	Possible, on volumetric rendering or good midsagittal view of the fetal face [18,19,20,21,22,23,24,25,26,27,28,29,30,31,32]
Long and/or smooth philtrum	Possible, on volumetric rendering or good midsagittal view of the fetal face [18,19,20,21,22,23,24,25,26,27,28,29,30,31,32]
Thin upper lip vermilion and/or downturned corners of mouth	Possible with volumetric ultrasound [21,32]
Hand oligodactyly and/or adactyly	Possible; mandatory if severe defect [20,21,22,30,31,32]
Congenital diaphragmatic hernia	Possible in most cases [22,28,31,32]
Suggestive features *	
Global developmental delay and/or intellectual disability	Postnatal diagnosis
Prenatal growth retardation (<2SD)	Yes [18,21,22,23,24,31,32,33]
Postnatal growth retardation (<2SD)	Postnatal diagnosis
Microcephaly	Yes [18,22,23,24,31,33]
Small hands and/or feet	Difficult to objectively assess, very few reports of prenatal diagnosis [31]
Short fifth finger	Yes [21,22]
Hirsutism	Difficult prenatal diagnosis [21,22]

* Cardinal and suggestive features of CDLS were described in a Delphi consensus statement in 2018 [2].

## References

[1] Deardorff M.A., Noon S.E., Krantz I.D., Adam M.P., Ardinger H.H., Pagon R.A., Wallace S.E., Bean L.J.H., Stephens K., Amemiya A. (1993–2020). Cornelia de Lange Syndrome 2020 October 15. Gene Reviews^®^ [Internet].

[2] Kline A.D., Moss J.F., Selicorni A., Bisgaard A.M., Deardorff M.A., Gillett P.M., Ishman S.L., Kerr L.M., Levin A.V., Mulder P.A. (2018). Diagnosis and management of Cornelia de Lange syndrome: First international consensus statement. Nat. Rev. Genet..

[3] Avagliano L., Parenti I., Grazioli P., Di Fede E., Parodi C., Mariani M., Kaiser F.J., Selicorni A., Gervasini C., Massa V. (2020). Chromatinopathies: A focus on Cornelia de Lange syndrome. Clin. Genet..

[4] Kline A.D., Grados M., Sponseller P., Levy H.P., Blagowidow N., Schoedel C., Rampolla J., Clemens D.K., Krantz I., Kimball A. (2007). Natural history of aging in Cornelia de Lange syndrome. Am. J. Med. Genet. C Semin. Med. Genet..

[5] Jackson L., Kline A.D., Barr M.A., Koch S. (1993). De Lange syndrome: A clinical review of 310 individuals. Am. J. Med. Genet..

[6] Boyle M.I., Jespersgaard C., Brøndum-Nielsen K., Bisgaard A.M., Tümer Z. (2015). Cornelia de Lange syndrome. Clin. Genet..

[7] Cascella M., Muzio M.R. (2020). Cornelia de Lange Syndrome 4 July 2020. Stat Pearls [Internet].

[8] Barisic I., Tokic V., Loane M., Bianchi F., Calzolari E., Garne E., Wellesley D., Dolk H., EUROCAT Working Group (2008). Descriptive epidemiology of Cornelia de Lange syndrome in Europe. Am. J. Med. Genet. A.

[9] Bhuiyan Z.A., Klein M., Hammond P., van Haeringen A., Mannens M.M., Van Berckelaer-Onnes I., Hennekam R.C. (2006). Genotype-phenotype correlations of 39 patients with Cornelia De Lange syndrome: The Dutch experience. J. Med. Genet..

[10] Yan J., Saifi G.M., Wierzba T.H., Withers M., Bien-Willner G.A., Limon J., Stankiewicz P., Lupski J.R., Wierzba J. (2006). Mutational and genotype-phenotype correlation analyses in 28 Polish patients with Cornelia de Lange syndrome. Am. J. Med. Genet. A.

[11] Selicorni A., Russo S., Gervasini C., Castronovo P., Milani D., Cavalleri F., Bentivegna A., Masciadri M., Domi A., Divizia M.T. (2007). Clinical score of 62 Italian patients with Cornelia de Lange syndrome and correlations with the presence and type of NIPBL mutation. Clin. Genet..

[12] Mariani M., Decimi V., Bettini L.R., Maitz S., Gervasini C., Masciadri M., Ajmone P., Kullman G., Dinelli M., Panceri R. (2016). Adolescents and adults affected by Cornelia de Lange syndrome: A report of 73 Italian patients. Am. J. Med. Genet. C Semin. Med. Genet..

[13] Cereda A., Mariani M., Rebora P., Sajeva A., Ajmone P.F., Gervasini C., Russo S., Kullmann G., Valsecchi G., Selicorni A. (2016). A new prognostic index of severity of intellectual disabilities in Cornelia de Lange syndrome. Am. J. Med. Genet. C Semin. Med. Genet..

[14] Pié J., Puisac B., Hernández-Marcos M., Teresa-Rodrigo M.E., Gil-Rodríguez M., Baquero-Montoya C., Ramos-Cáceres M., Bernal M., Ayerza-Casas A., Bueno I. (2016). Special cases in Cornelia de Lange syndrome: The Spanish experience. Am. J. Med. Genet. C Semin. Med. Genet..

[15] Hei M., Gao X., Wu L. (2018). Clinical and genetic study of 20 patients from China with Cornelia de Lange syndrome. BMC Pediatr..

[16] Liu C., Li X., Cui J., Dong R., Lv Y., Wang D., Zhang H., Li X., Li Z., Ma J. (2020). Analysis of clinical and genetic characteristics in 10 Chinese individuals with Cornelia de Lange syndrome and literature review. Mol. Genet. Genom. Med..

[17] Dowsett L., Porras A.R., Kruszka P., Davis B., Hu T., Honey E., Badoe E., Thong M.K., Leon E., Girisha K.M. (2019). Cornelia de Lange syndrome in diverse populations. Am. J. Med. Genet. A.

[18] Ranzini A.C., Day-Salvatore D., Farren-Chavez D., McLean D.A., Greco R. (1997). Prenatal diagnosis of de Lange syndrome. J. Ultrasound Med..

[19] Sepulveda W., Wong A.E., Dezerega V. (2009). Brachmann-de Lange Syndrome: Prenatal diagnosis with 2- and 3-dimensional sonography. J. Ultrasound Med..

[20] Kanellopoulos V., Iavazzo C., Tzanatou C., Papadakis E., Tassis K. (2011). A case of third trimester diagnosis of Cornelia de Lange syndrome. Arch. Gynecol. Obstet..

[21] Thellier E., Levaillant J.M., Roume J., Quarello E., Bault J.P. (2017). Cornelia de Lange syndrome: Specific features for prenatal diagnosis. Ultrasound Obstet. Gynecol..

[22] Avagliano L., Bulfamante G.P., Massa V. (2017). Cornelia de Lange syndrome: To diagnose or not to diagnose in utero?. Birth Defects Res..

[23] Manouvrier S., Espinasse M., Vaast P., Boute O., Farre I., Dupont F., Puech F., Gosselin B., Farriaux J.P. (1996). Brachmann-de Lange syndrome: Pre- and postnatal findings. Am. J. Med. Genet..

[24] Boog G., Sagot F., Winer N., David A., Nomballais M.F. (1999). Brachmann-de Lange syndrome: A cause of early symmetric fetal growth delay. Eur. J. Obstet. Gynecol. Reprod. Biol..

[25] Sekimoto H., Osada H., Kimura H., Kamiyama M., Arai K., Sekiya S. (2000). Prenatal findings in Brachmann-de Lange syndrome. Arch. Gynecol. Obstet..

[26] Urban M., Hartung J. (2001). Ultrasonographic and clinical appearance of a 22-week-old fetus with Brachmann-de Lange syndrome. Am. J. Med. Genet..

[27] Huang W.H., Porto M. (2002). Abnormal first-trimester fetal nuchal translucency and Cornelia De Lange syndrome. Obstet. Gynecol..

[28] Marino T., Wheeler P.G., Simpson L.L., Craigo S.D., Bianchi D.W. (2002). Fetal diaphragmatic hernia and upper limb anomalies suggest Brachmann-de Lange syndrome. Prenat. Diagn..

[29] Chong K., Keating S., Hurst S., Summers A., Berger H., Seaward G., Martin N., Friedberg T., Chitayat D. (2009). Cornelia de Lange syndrome (CdLS): Prenatal and autopsy findings. Prenat. Diagn..

[30] Wilmink F.A., Papatsonis D.N., Grijseels E.W., Wessels M.W. (2009). Cornelia de Lange syndrome: A recognizable fetal phenotype. Fetal. Diagn. Ther..

[31] Pajkrt E., Griffin D.R., Chitty L.S. (2010). Brachmann-de Lange syndrome: Definition of prenatal sonographic features to facilitate definitive prenatal diagnosis. Prenat. Diagn..

[32] Clark D.M., Sherer I., Deardorff M.A., Byrne J.L., Loomes K.M., Nowaczyk M.J., Jackson L.G., Krantz I.D. (2012). Identification of a prenatal profile of Cornelia de Lange syndrome (CdLS): A review of 53 CdLS pregnancies. Am. J. Med. Genet. A.

[33] Kliewer M.A., Kahler S.G., Hertzberg B.S., Bowie J.D. (1993). Fetal biometry in the Brachmann-de Lange syndrome. Am. J. Med. Genet..

[34] Bruner J.P., Hsia Y.E. (1990). Prenatal findings in Brachmann-de Lange syndrome. Obstet. Gynecol..

[35] Drolshagen L.F., Durmon G., Berumen M., Burks D.D. (1992). Prenatal ultrasonographic appearance of “Cornelia de Lange” syndrome. J. Clin. Ultrasound.

[36] Spaggiari E., Vuillard E., Khung-Savatovsky S., Muller F., Oury J.F., Delezoide A.L., Guimiot F. (2013). Ultrasound detection of eyelashes: A clue for prenatal diagnosis of Cornelia de Lange syndrome. Ultrasound Obstet. Gynecol..

[37] Dempsey M.A., Knight Johnson A.E., Swope B.S., Moldenhauer J.S., Sroka H., Chong K., Chitayat D., Briere L., Lyon H., Palmer N. (2014). Molecular confirmation of nine cases of Cornelia de Lange syndrome diagnosed prenatally. Prenat. Diagn..

[38] Hague J., Twiss P., Mead Z., Park S.M. (2019). Clinical Diagnosis of Classical Cornelia de Lange Syndrome Made from Postmortem Examination of Second Trimester Fetus With Novel NIPBL Pathogenic Variant. Pediatr. Dev. Pathol..

[39] Richards S., Aziz N., Bale S., Bick D., Das S., Gastier-Foster J., Grody W.W., Hegde M., Lyon E., Spector E. (2015). Standards and guidelines for the interpretation of sequence variants: A joint consensus recommendation of the American College of Medical Genetics and Genomics and the Association for Molecular Pathology. Genet. Med..

[40] Nedelea F., Veduta A., Duta S., Vayna A.M., Panaitescu A., Peltecu G., Duba H.C. (2018). Prenatal Genetic Testing for Dopa-Responsive Dystonia—Clinical Judgment in the Context of Next Generation Sequencing. J. Med. Life.

[41] Huisman S.A., Redeker E.J., Maas S.M., Mannens M.M., Hennekam R.C. (2013). High rate of mosaicism in individuals with Cornelia de Lange syndrome. J. Med. Genet..

[42] Ansari M., Poke G., Ferry Q., Williamson K., Aldridge R., Meynert A.M., Bengani H., Chan C.Y., Kayserili H., Avci S. (2014). Genetic heterogeneity in Cornelia de Lange syndrome (CdLS) and CdLS-like phenotypes with observed and predicted levels of mosaicism. J. Med. Genet..

[43] Mannini L., Cucco F., Quarantotti V., Krantz I.D., Musio A. (2013). Mutation spectrum and genotype-phenotype correlation in Cornelia de Lange syndrome. Hum. Mutat..

[44] Pié J., Gil-Rodríguez M.C., Ciero M., López-Viñas E., Ribate M.P., Arnedo M., Deardorff M.A., Puisac B., Legarreta J., de Karam J.C. (2010). Mutations and variants in the cohesion factor genes NIPBL, SMC1A, and SMC3 in a cohort of 30 unrelated patients with Cornelia de Lange syndrome. Am. J. Med. Genet. A.

[45] Schoumans J., Wincent J., Barbaro M., Djureinovic T., Maguire P., Forsberg L., Staaf J., Thuresson A.C., Borg A., Nordgren A. (2007). Comprehensive mutational analysis of a cohort of Swedish Cornelia de Lange syndrome patients. Eur. J. Hum. Genet..

[46] Tonkin E.T., Wang T.J., Lisgo S., Bamshad M.J., Strachan T. (2004). NIPBL, encoding a homolog of fungal Scc2-type sister chromatid cohesion proteins and fly Nipped-B, is mutated in Cornelia de Lange syndrome. Nat. Genet..

[47] Liu J., Krantz I.D. (2009). Cornelia de Lange syndrome, cohesin, and beyond. Clin. Genet..

[48] Sarogni P., Pallotta M.M., Musio A. (2020). Cornelia de Lange syndrome: From molecular diagnosis to therapeutic approach. J. Med. Genet..

[49] Borck G., Redon R., Sanlaville D., Rio M., Prieur M., Lyonnet S., Vekemans M., Carter N.P., Munnich A., Colleaux L. (2004). NIPBL mutations and genetic heterogeneity in Cornelia de Lange syndrome. J. Med. Genet..

[50] Gillis L.A., McCallum J., Kaur M., DeScipio C., Yaeger D., Mariani A., Kline A.D., Li H.H., Devoto M., Jackson L.G. (2004). NIPBL mutational analysis in 120 individuals with Cornelia de Lange syndrome and evaluation of genotype-phenotype correlations. Am. J. Hum. Genet..

[51] Deardorff M.A., Wilde J.J., Albrecht M., Dickinson E., Tennstedt S., Braunholz D., Mönnich M., Yan Y., Xu W., Gil-Rodríguez M.C. (2012). RAD21 mutations cause a human cohesinopathy. Am. J. Hum. Genet..

[52] Gudmundsson S., Annerén G., Marcos-Alcalde Í., Wilbe M., Melin M., Gómez-Puertas P., Bondeson M.L. (2019). A novel RAD21 p. (Gln592del) variant expands the clinical description of Cornelia de Lange syndrome type 4—Review of the literature. Eur. J. Med. Genet..

[53] Krab L.C., Marcos-Alcalde I., Assaf M., Balasubramanian M., Andersen J.B., Bisgaard A.M., Fitzpatrick D.R., Gudmundsson S., Huisman S.A., Kalayci T. (2020). Delineation of phenotypes and genotypes related to cohesin structural protein RAD21. Hum. Genet..

[54] Deardorff M.A., Kaur M., Yaeger D., Rampuria A., Korolev S., Pie J., Gil-Rodríguez C., Arnedo M., Loeys B., Kline A.D. (2007). Mutations in cohesin complex members SMC3 and SMC1A cause a mild variant of Cornelia de Lange syndrome with predominant mental retardation. Am. J. Hum. Genet..

[55] Gil-Rodríguez M.C., Deardorff M.A., Ansari M., Tan C.A., Parenti I., Baquero-Montoya C., Ousager L.B., Puisac B., Hernández-Marcos M., Teresa-Rodrigo M.E. (2015). De novo heterozygous mutations in SMC3 cause a range of Cornelia de Lange syndrome-overlapping phenotypes. Hum. Mutat..

[56] Olley G., Ansari M., Bengani H., Grimes G.R., Rhodes J., von Kriegsheim A., Blatnik A., Stewart F.J., Wakeling E., Carroll N. (2018). BRD4 interacts with NIPBL and BRD4 is mutated in a Cornelia de Lange-like syndrome. Nat. Genet..

[57] Parenti I., Gervasini C., Pozojevic J., Graul-Neumann L., Azzollini J., Braunholz D., Watrin E., Wendt K.S., Cereda A., Cittaro D. (2016). Broadening of cohesinopathies: Exome sequencing identifies mutations in ANKRD11 in two patients with Cornelia de Lange-overlapping phenotype. Clin. Genet..

[58] Deardorff M.A., Porter N.J., Christianson D.W. (2016). Structural aspects of HDAC8 mechanism and dysfunction in Cornelia de Lange syndrome spectrum disorders. Protein Sci..

[59] Kaiser F.J., Ansari M., Braunholz D., Concepción Gil-Rodríguez M., Decroos C., Wilde J.J., Fincher C.T., Kaur M., Bando M., Amor D.J. (2014). Loss-of-function HDAC8 mutations cause a phenotypic spectrum of Cornelia de Lange syndrome-like features.; ocular hypertelorism.; large fontanelle and X-linked inheritance. Hum. Mol. Genet..

[60] Parenti I., Gervasini C., Pozojevic J., Wendt K.S., Watrin E., Azzollini J., Braunholz D., Buiting K., Cereda A., Engels H. (2016). Expanding the clinical spectrum of the ‘HDAC8-phenotype’—Implications for molecular diagnostics, counseling and risk prediction. Clin. Genet..

[61] Musio A., Selicorni A., Focarelli M.L., Gervasini C., Milani D., Russo S., Vezzoni P., Larizza L. (2006). X-linked Cornelia de Lange syndrome owing to SMC1L1 mutations. Nat. Genet..

[62] Borck G., Zarhrate M., Bonnefont J.P., Munnich A., Cormier-Daire V., Colleaux L. (2007). Incidence and clinical features of X-linked Cornelia de Lange syndrome due to SMC1L1 mutations. Hum. Mutat..

[63] Huisman S., Mulder P.A., Redeker E., Bader I., Bisgaard A.M., Brooks A., Cereda A., Cinca C., Clark D., Cormier-Daire V. (2017). Phenotypes and genotypes in individuals with SMC1A variants. Am. J. Med. Genet. A.

[64] Woods S.A., Robinson H.B., Kohler L.J., Agamanolis D., Sterbenz G., Khalifa M. (2014). Exome sequencing identifies a novel EP300 frame shift mutation in a patient with features that overlap Cornelia de Lange syndrome. Am. J. Med. Genet. A.

[65] Yuan B., Pehlivan D., Karaca E., Patel N., Charng W.L., Gambin T., Gonzaga-Jauregui C., Sutton V.R., Yesil G., Bozdogan S.T. (2015). Global transcriptional disturbances underlie Cornelia de Lange syndrome and related phenotypes. J. Clin. Investig..

[66] Vayna A.M., Veduta A., Duta S., Panaitescu A.M., Stoica S., Buinoiu N., Nedelea F., Peltecu G. (2018). Diagnosis of Fetal Structural Anomalies at 11 to 14 Weeks. J. Ultrasound Med..

